# A deep learning framework for non-functional requirement classification

**DOI:** 10.1038/s41598-024-52802-0

**Published:** 2024-02-08

**Authors:** Kiramat Rahman, Anwar Ghani, Sanjay Misra, Arif Ur Rahman

**Affiliations:** 1 Department of Software Engineering, International Islamic University, Islamabad, 44000 Pakistan; 2 Department of Computer Science, International Islamic University, Islamabad, 44000 Pakistan; 3 Department of Computer Science and Communication, Østfold University College, Halden, Norway; 4 Department of Applied Data Science, Institute for Energy Technology, Halden, Norway; 5 Department of Computer Science, Bahria University, Islamabad, 44000 Pakistan

**Keywords:** Computer science, Software

## Abstract

Analyzing, identifying, and classifying nonfunctional requirements from requirement documents is time-consuming and challenging. Machine learning-based approaches have been proposed to minimize analysts’ efforts, labor, and stress. However, the traditional approach of supervised machine learning necessitates manual feature extraction, which is time-consuming. This study presents a novel deep-learning framework for NFR classification to overcome these limitations. The framework leverages a more profound architecture that naturally captures feature structures, possesses enhanced representational power, and efficiently captures a broader context than shallower structures. To evaluate the effectiveness of the proposed method, an experiment was conducted on two widely-used datasets, encompassing 914 NFR instances. Performance analysis was performed on the applied models, and the results were evaluated using various metrics. Notably, the DReqANN model outperforms the other models in classifying NFR, achieving precision between 81 and 99.8%, recall between 74 and 89%, and F1-score between 83 and 89%. These significant results highlight the exceptional efficacy of the proposed deep learning framework in addressing NFR classification tasks, showcasing its potential for advancing the field of NFR analysis and classification.

## Introduction

Non-functional software requirements (NFR) play a crucial role and define as software system constraints and quality expectations^1–3^. However, the identification and classification of NFR often face challenges due to its inherited nature and various other reasons^4–6^. To begin with financial and technical constraints, developers often overlook the significance of NFRs^7,8^, pay them less attention^9^, or delay their implementation until late in the development process^10^. In addition, NFRs are subjective and scattered across requirement specification artifacts^10,11^, making their identification and consolidation a tedious, error-prone, and time-consuming process^12,13^. Furthermore, manual identification and classification of NFR require significant human effort, leading to the need for automated approaches^14^. To address these challenges, various machine learning-based approaches have been proposed to automate the identification and classification of NFRs^15^. However, the performance of ML-based methods depends on the availability and quality of the dataset, which poses additional challenges in the field of requirement engineering^16^. Traditional ML-based methods rely on handcrafted features to classify NFR, which is time-consuming and error-prone. In addition, when the feature space changes, the performance of these methods is degraded^17,18^. On the other hand, Deep Neural Networks (DNN) offer the potential to extract features from the requirements documents automatically. In addition, DNNs have shown promising results in various domains, including natural language processing, image processing, and speech recognition^19^.

Past studies such as Kurtanovic^20^, Agustin Casamayor^21^, and Eric Knauss^22^ have proposed different approaches for software requirement classification. Although these approaches have promising results, manual feature selection makes them less practical. To overcome this issue, this study integrated advanced techniques, such as optimization and regularization strategies, with a multilayer architecture comprising DReqANN and DReqBiLSTM models. Compared to Cody Backer et al.^23^, the single-layer ANN approach leads to instability in the multiclass classification of NFR.In contrast, this study proposed comprehensive frameworks with a two-phase model construction, demonstrating improved stability and enhanced classification capabilities for multi-class nonfunctional requirements. Moreover, the proposed framework includes preprocessing and feature extraction phases, which collectively contribute to the robustness and efficiency of the NFR classification process.

Recently, Hey et al.^24^, Li and Nong^25^, and Li et al.^16^ have conducted a study using complex large language models for the classification of software requirements. However, these approaches require extensive computational resources, and the initialization of large models with a small dataset leads to instability in performance. On the other hand, the proposed approach in this study is resource-efficient, making it suitable for smaller datasets.The motivation behind this study is to overcome the limitations of the traditional ML-based approach for NFR classification and empower the process with deep learning techniques. The objectives of this article are:

- To investigate the effectiveness and suitability of deep neural networks and shallower neural networks in NFR classification tasks, particularly in scenarios where the availability of the dataset is limited.
- To automatically extract features from requirements documents leveraging the capabilities of deep learning techniques to optimize the NFR classification process and improve overall efficiency.
- To eliminate the need for handcrafted feature selection.
- To assess and improve the performance of DNN in NFR classification with limited available datasets.

This study has proposed a framework for classifying multi-class non-functional requirements using deep learning approaches to achieve these objectives. The proposed framework eliminates the need for handcrafted features, providing a more automated and efficient approach. The main contribution of this study is the development of the DReqANN and DReqBiLSTM deep learning models tailored explicitly for NFR classification. These models utilize advanced techniques such as word embedding, optimization and regularization techniques, and multi-layer architecture to classify NFRs effectively. The effectiveness of these models is demonstrated through rigorous empirical evaluation, comparing the results with different statistical measurements. To validate the proposed framework, an experiment has been conducted using publicly available datasets, ensuring the reliability and reproducibility of the results. The experimental findings demonstrate the effectiveness of the proposed framework in accurately classifying NFRs.Overall, this study contributes to the field of NFR classification by offering a comprehensive framework, novel deep-learning models, improved accuracy, and empirical validation. The general contributions of this study are:

- Proposes a framework for classifying multi-class non-functional requirements to support analysts and developers without handcrafted features.
- Develops a novel deep learning model architecture DReqANN and DReqBiLSTM specifically tailored for NFR classification.
- Presents the interpretation and analysis of the classifiers’ performance, offering insights into the underlying mechanisms of NLP and machine learning.
- Validate the proposed framework through rigorous experimentations using publicly available data sets, ensuring the reliability and reproducibility of the results.
- Comparatively analyzed the proposed approach with state-of-the-art approaches in terms of different evaluation metrics.

The rest of this paper is organized as follows. Section “Related work” presents related works. Section “Proposed approach” introduces the proposed approach, while section “Model Evaluation Phase” describes the experimental setup and analysis of the results. Section “Threats to validity” identifies threats to validity, and section “Conclusion” concludes the article.

## Related work

Over the past years, several researchers have classified non-functional requirements (NFRs). While some of these works have identified many NFR categories, others have focused on developing automated approaches for NFR classification. This section reviews some of the most relevant works in this area.

Chung et al.^14^ performed a comprehensive classification of NFRs and identified almost 150 categories of NFRs. Roman^26^ introduced six constraints of NFRs related to economics, interface, life-cycle, operating, performance, and politics. Slankas and Williams^27^ analyzed different requirements documents and defined 14 categories of NFRs. There needs to be more consensus on the categorization of NFR, leading to different interpretations or categorizations. Additionally, classifying NFRs is a complex activity requiring domain knowledge. The categorization of NFR may be influenced by the analyst’s experience and expertise, which may not be replicable by another analyst with different expertise and experience. The categorization of NFR is based on expert opinion and perception and has no empirical validation, and they do not reflect the actual NFRs encountered in real-world projects. While the manual classification of NFRs is subject to errors due to the domain expertise of the software engineer, various automated approaches have been introduced to classify NFRs automatically. Cleland-Huang et al.^10^ used information retrieval techniques to detect and classify NFRs from structured and free-form requirements documents. In this approach, natural language processing techniques extract a possible set of NFRs, which domain experts manually evaluate to identify the correct ones. The approach handles both structured and unstructured data, making it more practical than other approaches. However, the manual validation of NFR is time-consuming and error-prone, reducing the efficiency and accuracy of this approach. Lu and Liang^28^ classified NFRs into four types (reliability, usability, portability, and performance) using machine learning algorithms from user application reviews. They used Naive Bayes, J48, and Bagging combined with four classification techniques: Bag of Words (BoW), TF-IDF, CHI2, and AUR-BoW. This approach has overcome the manual intervention of the previous approach by using a large amount of data to classify NFR automatically. However, the quality and representativeness of the data used for training may hurt the generalization of this approach to other contexts or domains. Kurtanovic^20^ proposed a supervised learning approach using a Support Vector Machine (SVM) with lexical features, achieving good results for binary classification of NFRs but could have performed better on multi-class classifications. This approach is simple and efficient in handling the binary classification of NFRs. However, more complex algorithms and automatic feature extraction techniques are required to improve the performance and accuracy of multiclass classification of NFR.

Gokyer et al. ^29^ have deployed SVM to identify NFR and map it to architectural concerns. However, the manual refinement of the knowledge base repository and the inability of SVM to capture complex relationships and patterns within data may limit its scalability and effectiveness. Agustin Casamayor^21^ proposed a semi-supervised approach to identify NFR using the Expectation Maximization (EM) strategy and naive Bayesian algorithms. However, the semi-supervised approach increased complexity and effort with marginal gain in accuracy. In addition, the initially labeled dataset and skewed distribution of labeled and unlabeled data decreased NFR classification accuracy. Eric Knauss^22^ has used a Bayesian classifier to identify security-related requirements; however, this approach has limited generalizability to other non-functional requirements. Slankas^27^ presented the NFR Locator process and tool for extraction of NFR; however, the study used a traditional ML method that relies on handcrafted features, which is a time-consuming and subjective process. In addition, the model becomes less effective when feature space changes or significant retraining is required.

Dekhtyar and Fong^30^ have implemented word2vec embedding and Convolutional Neural Network(CNN) to classify NFRs. This study has performed binary classification and did not perform multi-class classification of NFRs. Cody Backer et al.^23^ have proposed a similar approach for NFR classification but have implemented single-layer ANN, and they have classified NFR into four classes due to the instability of the model. At the same time, CNN has promising results in handling computer vision but cannot handle text and audio. While classifying sequential text, CNN cannot preserve representations from previous input data during the learning process. This can lead to problems in classifying sequential text where a significant relationship exists between the last set of statements. RNN has addressed this issue of why RNN is the de facto method for modeling text data. NFR is a sequential classification, so LSTM and BiLSTM are better candidates for NFR classifications.

Rahman et al.^31^ proposed an approach using RNN architecture LSTM and GRU Variants. The data set used in this study consists of 370 NFRs, which needs to be revised for complex neural networks and may not result in reliable trained models. This study rectifies this issue by combining two publicly available data sets containing 1165 NFRs. Comparative analysis of the related works is presented in Table 1.

Various approaches to the classification of NFR have been presented in the existing literature; however, these methods suffer from inherited challenges. Numerous studies have highlighted the manual classification challenges, such as potential error and subjectivity injected through individual analysis and perception. The emergence of the traditional ML-based method for NFR classification has inherited the problem of manual feature selection that resulted in performance degradation when applied to different sets of features. On the other hand, deep learning models offer automatic feature selection, overcoming the limitations of traditional ML-based approaches, but their dependency on large datasets presents a significant obstacle. Specifically, the lack of supervised data is a prevailing issue in software requirement engineering. Training a DNN model with a small dataset results in overfitting issues. Thus, it is relevant to address these challenges. This study has proposed a novel deep-learning framework to overcome the limitations of traditional ML-based methods for NFR classifications. The proposed framework aims to tackle the advantages of deep learning while mitigating overfitting concerns in a limited dataset. This study has presented a more reliable and adaptable classification approach that empowers analysts to make informed decisions in developing robust and adaptable software solutions.

**Table 1:** Summary of related works.

Papers	Feature extraction methods	Modeling Approaches/Algorithms	Limitation
Cleland-Huang et al.^10^	NLP with expert evaluations	Information retrieval techniques	Manual evaluation is time-consuming.
Lu and Liang^28^	BoW,TFIDF,CHI2,and AUR-BoW	Naive Bayes, J48, and Bagging	Manual feature selection and handling dimensionality of data is challenging
Kurtanovic^20^	Lexical feature extraction	Support Vector Machine(SVM)	Manual feature selection is required
Gokyer et al.^29^	POS taging	SVM	SVM does not capture complex relationship patterns within the data to predict and classify NFR efficiently
Agustin Casamayor^21^	Vector space model (VSM)	Expectation Maximization and naive Bayesian algorithms	Semi-supervised method increases complexity and skewed distribution resulting in performance degradation
Slankas, J^27^	Word2Vec	NFR Locator process	Handcrafted features are time-consuming. The model becomes less effective when the feature space changes
Dekhtyar and Fong^30^	Word2Vec, TFIDF	CNN	Limited to a binary classification of requirements
Cody Backer et al.^23^	BoW	ANN and CNN	The instability of the ANN model and CNN do not preserve representation from previous input data
Rahman et al.^31^	Word2Vec	RNN and LSTM	Reliability and overfitting of the model due to a small dataset with 370 NFR

## Proposed approach

**Figure 1:**
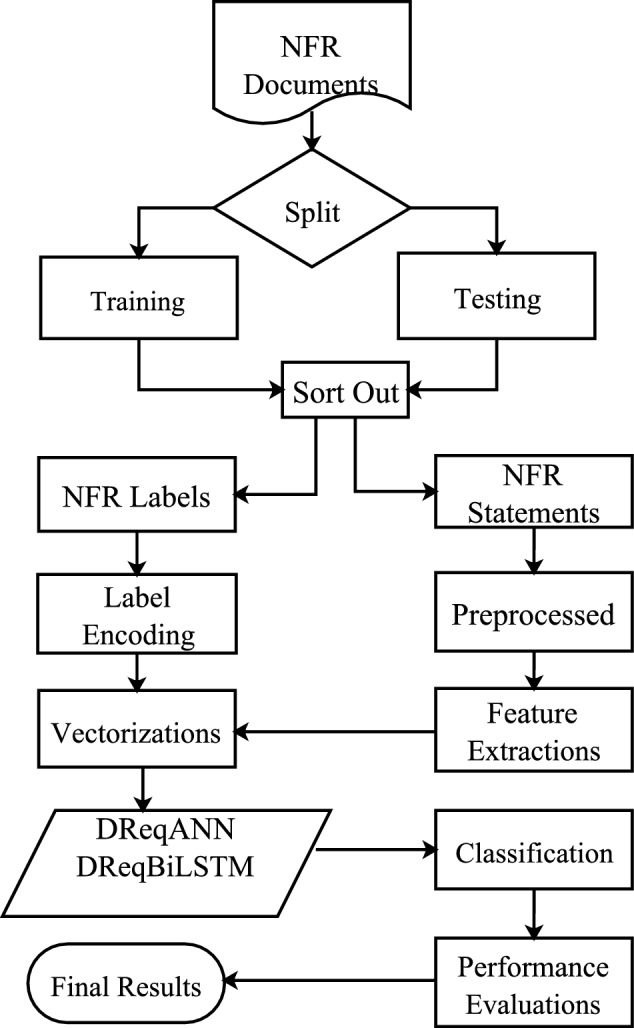
General flow of the proposed methods.

Figure 1 illustrates the overall framework of Multi-class NFR classification that takes requirement documents as input and outputs their respective categorization. The framework consists of four phases: 1) the Preprocessing Phase, 2) the Feature Extraction and Word Vectorization Phase, 3) the Model Construction Phase, and 4) the Model Training and Evaluation Phase. Phase 3) consists of two sub-phases: DReqANN and DReqBiLSTM.

The code and data used in this study are available on the GitHub repository^32^. The following subsection elaborately describes the above step in detail.

### Preprocessing phase

The preprocessing phase includes several techniques selected based on their effectiveness in NFR classifications. In addition, it also ensures their compatibility with the proposed deep neural network architecture. Furthermore, these techniques improve the models’ accuracy and reduce resource consumption and computation time.

In the first step, we apply case folding to unify the requirements description to eliminate redundancy and avoid potential confusion in the subsequent classification process. To improve the performance of models for software requirement classification, various elements that may degrade the quality of the input data are removed, such as punctuation marks, company names, hashtags, and other symbols. In addition, custom words that are found irrelevant for NFR classification are manually identified and eliminated. This process led to improved model performance with a limited data set because the model focuses on meaningful content words and reduces noise that may interfere with the accurate classification of NFR.

Frequent and commonly occurring words with little or no semantic meaning are removed using Natural Language Toolkit (NLTK) libraries. These words do not contribute useful information for NFR classification. This process reduces the dimensionality of the data and eliminates unnecessary noise. Word net lemmatization converts words into their base or root form to handle word variations and maintain accuracy. Lemmatization supports clarity, avoids potential ambiguity, ensures consistent representation, and facilitates accurate NFR classifications.

Tokenization is a crucial preprocessing step for extracting meaningful features from text data. This study used Word tokenization algorithms to split the requirement statement into individual tokens. These tokens are further used to create vocabulary and assign unique identifiers to represent words in numerical format. To overcome the Out of Vocabulary (OOV) issues during testing, this study introduced a special “<UKN>” token. To maintain the integrity of the NFR classification process, any word not found in the vocabulary is replaced with this unknown token. At the end of the preprocessing steps, the requirement documents are fed to the next phase for conversion into a machine-readable format.

### Feature extraction and word vectorization phase

In this phase, the feature retrieved from the requirement document is transformed into vector form, allowing machine learning models to process and learn efficiently and effectively. This conversion facilitates the proposed DReqANN and DReqBiLSTM models to successfully handle and learn from requirements data. These models use numerical representations to extract meaningful patterns, identify significant characteristics, and create accurate predictions for NFR categorization. Different techniques are employed to transform features into vector form. This study uses TF-IDF and word embedding techniques to convert requirement features into vector form.

#### Term frequency-inverse document frequency (TF-IDF)

TF-IDF extracts features and converts requirement documents into numerical representations. Specifically, TF-IDF is beneficial for NFR classification as this technique captures the relative importance of terms within individual requirement documents and across all requirement documents. Both TF (1) and IDF (2) components are combined,

1 $$\begin{aligned}{} & {} tf(t,d)=\log {(1+frq)(t,d)} \end{aligned}$$

2 $$\begin{aligned}{} & {} idf(t,D)=\log \bigg ( {\frac{N}{count(d\in D:t\in d)}}\bigg ) \end{aligned}$$

Then, the TF-IDF (3) assigns the higher weights to terms frequent in the requirement document but rare in the overall requirement corpus.

3 $$\begin{aligned} tfidf(t,d,D)=tf(t,d)*idf(t,D) \end{aligned}$$

This technique allows DreqANN to learn meaningful patterns and focus on discriminative terms for different NFR types, making an informed decision and enabling better NFR classification accuracy. TF-IDF matrix highlights essential terms and their relevance to NFR classifications, providing a rich representation of requirement documents.

#### Kerass word embedding

Understanding the meaning and context of requirements is crucial for NFR classifications. Therefore, this study used the Word Embedding technique. To capture the semantic relationship and contextual information among the words. Word embedding converts words into a dense vector and represents them in the vector space where the semantic similarities between words are preserved. This allows the model to capture the relationship among NFR words even if it is not explicitly mentioned in the training data. Word embedding enhanced the DReq-BiLSTM model’s capability to generalize and classify NFR based on the underlying semantics of the requirements.

### Models construction phase

The aim of this study is the accurate classification of nonfunctional software requirements using deep neural networks. To achieve this aim, two specific tailored models, Deep Requirement Artificial Neural Network (DReqANN) and Deep Requirement Bidirectional Long Short Term Memory (DReqBiLSTM) models, are developed and trained for NFR classifications. The reason behind proposing these architectures is the unique strength of DNN. In our case, the ANN-based architecture is suitable for learning complex patterns and relationships among the NFR documents by modeling nonlinear boundaries to classify NFR. In addition, it reduces the efforts of manual feature extraction, which is a time-consuming and challenging task in traditional ML-based methods. On the other hand, BiLSTM-based architecture is specifically tailored to capture contextual and sequential information of NFR data^33^.

#### DReq-ANN models architecture

**Figure 2:**
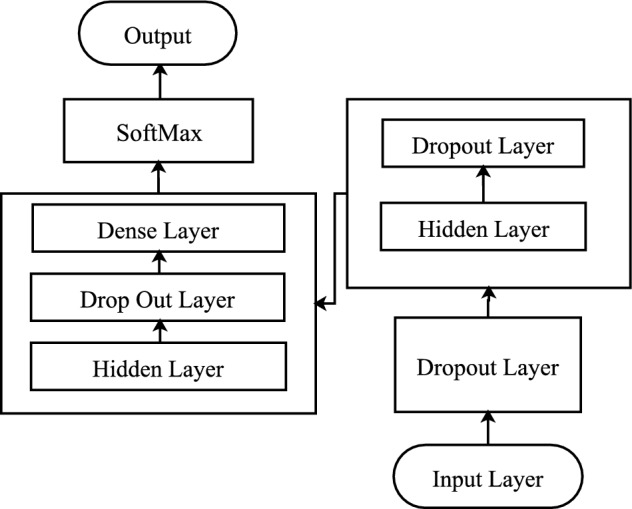
DReqANN model architecture.

Figure 2 illustrates the architecture of DReqANN, which consists of seven layers, including one(1) Input layer, two(2) hidden layers, three(3) dropout layers, and one (1) output layer.

The input layer receives the preprocessed data in the form of a vector. The input data is transformed into a matrix representation where the row represents the sample of NFR, and the column represents the feature. The number of units equals the total number of unique features with an additional one. The input dimension is the maximum sequence length of NFR documents. Further, the randomly initial weight and bias are added to each input during the interconnection of the input layer with hidden layers. A weighted sum is passed to the Relu activation function responsible for which node to fire for feature extraction. Finally, the processed data is passed to the following hidden layers.

Two hidden layers have been used that perform all sorts of computation, learn the word and context, and transfer the results to the output layer. At the hidden layer, the Relu (4) activation function is used because this function is computationally efficient.

4 $$\begin{aligned} f(x) = \max (0, x) \end{aligned}$$

The dropout layer reduces overfitting and improves the performance of the models. The dropout layer randomly drops units from the neural network during training. Units and incoming and outgoing connections are temporarily removed from the networks. A fixed probability p independent of other units is specified to retain units. As recommended by^34^, the optimal retention probability is closer to 1 than 0.5. But retention probability can be set in the range of 0–1, on which value the model’s performance can be improved and reduce overfitting. The dropout layer after the input layer has been set with $p = 0.2$, after the first hidden layer with $p = 0.7$, and after the second dropout layer with $p = 0.8$ after a hit and trial with a different value.

The output layer is responsible for predicting the final categories of the respective classes. This layer received input from the previously hidden layers and assigned the probability to each class of NFR. The probabilistic softmax function 5 is used at this layer that assigns the respective probability to each class ranging from 0 to 1, and the sum of all probability is equal to one. The neuron unit at this layer equals the number of classes; in our case, it is five, representing the number of NFR classes.

#### DReqBiLSTM models architecture

**Figure 3:**
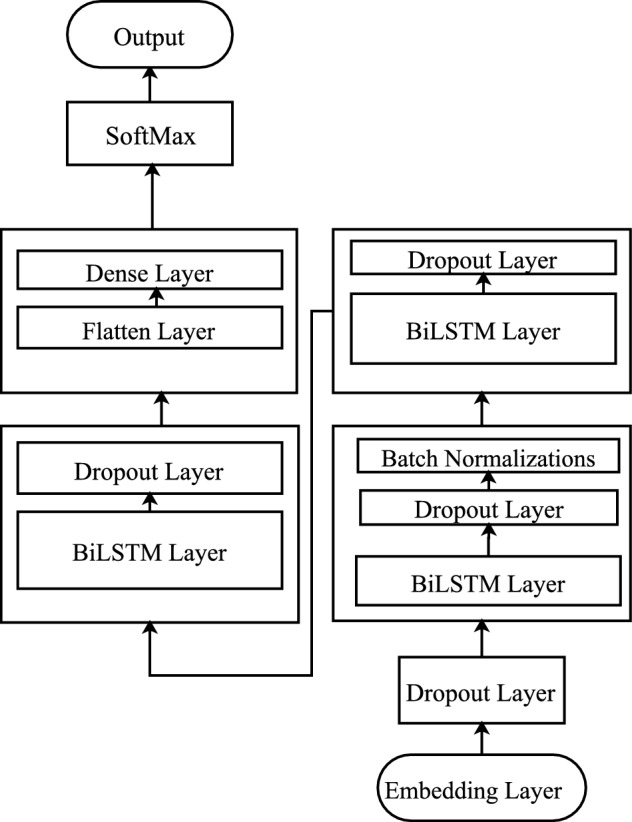
DReqBiLSTM model architecture.

Figure 3 presents the DReqBiLSTM architecture, which mainly consists of ten (10) layers, including one(1) embedding layer, three(3) hidden BiLSTM layers, one(1) Batch normalization layer, three dropout layers, one flatten layer, and one output layer.

The first layer is the embedding layer that takes the NFR padded sequence of word indices and converts it into an array of word vectors, a floating point number rather than an integer. Each word in the input NFR documents is represented by a vector in the embedding layer, and during the training, that is adjusted to reflect their relationship to each other in the embedding space. The parameter to the embedding layer was the length of vocabulary added with an additional one because indexing started with zero; otherwise, it causes an error. The second parameter is the number of dimensions; in our case, it is 100, and the third parameter is the max length of the padded sequence, which is 65, the maximum sequence length of a requirement statement in this study.

The hidden layer has used bidirectional LSTM (BiLSTM)^35^. The input to the bidirectional layer is the embedding layer’s output, a vector of the NFR documents. The features of vectors are extracted bidirectionally with the LSTM layer to produce the last sequence. Then, the previous sequence is classified in a dense layer with soft-max activation functions. The tanh activation function has been used in the hidden layer.

To stabilize the learning process and reduce the number of training epochs, batch normalization techniques^36^ have been used. Batch normalization accelerates the training, minimizes the generalization error, and improves the optimization.

Overfitting is an inherited problem of the deep neural-based method due to high complexity where the model learns noise in training data while considering it the actual underlying concept of the data. The dropout layer has been used in this study to reduce overfitting. During the model training, the dropout layer deactivates some input neurons with a random probability. The deactivated neuron is not a part of the neural network, resulting in a simple neural network with less complexity and less overfitting after the embedding layer dropout layer is added with $p= 0.2$. After the first BiLSTM layer, a Batch Normalization layer is added to normalize the data. After the second BiLSTM layer, we have added a Dropout layer with $p = 0.2$. After the third BiLSTM layer, we have added a Dropout layer with $p = 0.5$.

Flatten is the activity that converts the pooled feature to a solitary column fed to a fully connected layer. A flattened procedure reshapes a multi-dimensional matrix to a single dimension. The BiLSTM layer produces a multi-dimension matrix that a dense layer can not process. The flattened layer has been used to reshape a matrix into a vector form to be processed by a thick layer.

The softmax activation function was used with five units in the last dense layer. The softmax function transforms the output of the BiLSTM final layer into vector probabilities using the Eq. 5. The softmax function returns an output vector five entries long, the number of NFR classes used in this study. The total probabilities of all classes are in the range of zero to one.

5 $$\begin{aligned} \sigma (z_i) = \frac{e^{z_{i}}}{\sum _{j=1}^K e^{z_{j}}} \ \ \ for\ i=1,2,\dots ,K \end{aligned}$$

The class with maximum probability is selected as the respective class of NFR. To predict the label of NFRs, the argmax function is used on the output of softmax functions.

### Model training phase

Model configuration is required before training the models. Model configuration includes a loss function used to calculate the incorrectness of prediction and optimization algorithms used to minimize the error by updating network parameters. The loss function used in this study is categorical cross entropy 6 because it is a standard loss function for multi-class classification. The cross-entropy computed score is the average difference between actual and predicted probability distributions for all NFR classes.

6 $$\begin{aligned} H(x) = -\sum _{i=1}^M p(Y_i) \log _2 p(y) \end{aligned}$$

Where M is the number of classes, $p(Y_i)$ is the actual probability, and *p*(*y*) is the predicted probability. Adaptive moment estimation(Adam) is an optimization algorithm used in this study with an $\alpha = 0.001$, $\beta \_1 = 0.9$, $\beta \_2 = 0.999$, and $\epsilon = 1 \times 10^{-8}$. Batch size, sample number, and weight are updated once per training iteration. The training process ends with an epoch when the model has seen the entire training dataset. The validation dataset evaluates the model’s learning accuracy at the end of each epoch. This procedure is repeated for a fixed number of epochs.

One of the challenges of the training phase is the learning rate, which is weights updating during training in response to the estimated error. A large learning rate leads to unstable training, while tiny rates fail to train. The challenge of training the models involves careful selection of learning rate value. To address this issue, a technique is used to reduce the learning rate when there is no change for a given number of training epochs, hoping to fine-tune the models’ weight. The reduced learning rate on the plateau parameter is shown in Table 2, where the monitor is validation loss, monitored during training, and the learning rate is multiplied with factor and patience parameters.

Another challenge in training a machine learning model is how many epochs to run. More epochs may prevent model convergence, while excessive epochs may result in overfitting. Early stopping criteria have been used to stop training when the model starts to overfit without compromising the model’s accuracy. The model contains training when the validation error is no longer decreasing; in this way, the wastage of training resources is reduced. Early stopping not only reduces overfitting but also considers less number of epochs to train. Early stopping parameter and their value used in this study have been presented in Table 2. Where the monitor is validation loss, the patient is the number of epochs. The training is terminated if the monitor performance shows no more improvements for this number.

**Table 2:** Model training hyper parameters.

Models	Training hyper parameter			Call Back Criteria						
				Reduce on learning rate				Early stopping		
	Ephocs	Batch size	Verbose	Monitor	Factor	Patience	learning rate	Monitor	Min_delta	Patience
DReqANN	100	32	2	Val_loss	0.1	5	0.001	Val_loss	0	10
DReqBiLSTM	100	10	2	Val_loss	0.1	5	0.001	Val_loss	0	10

## Model Evaluation Phase

This section presents an evaluation of the proposed framework, including a brief description of the data set, experimental setup, and a detailed description of the evaluation metric.

### Dataset

This study used a publicly available dataset: the PROMIS-NFR available at^37^ and created by^10^ data set comprising requirements from 15 software projects. Whereas the expanded version PROMISE_exp available at^38^ and created by^39^ consists of 965 requirements from 47 requirement documents and the 2017 data challenge data set. All of these data sets consist of non-functional requirements. We combined all these data sets, resulting in 1165 unique NFRs falling into 14 categories. Since 9 of the 14 categories have a small number of samples, leading to an imbalanced dataset.

Training a DNN-based approach with an imbalanced dataset leads to several problems, including bias toward predicting the majority class more frequently and poor performance in accurately predicting the minority class. In addition, the imbalanced dataset with minority classes has fewer samples for training models, making it difficult to learn and generalize the pattern effectively, leading to poor performance and low recall for the minority class. Furthermore, During the training, DNN minimizes the overall loss on the training set to optimize the decision boundaries. In the case of an imbalanced dataset, the majority class influences the decision boundary, leading to poor separation between classes and lower precision for the minority class. In conclusion, DNN required a uniform data distribution during the training to learn the true underlying pattern.

**Figure 4:**
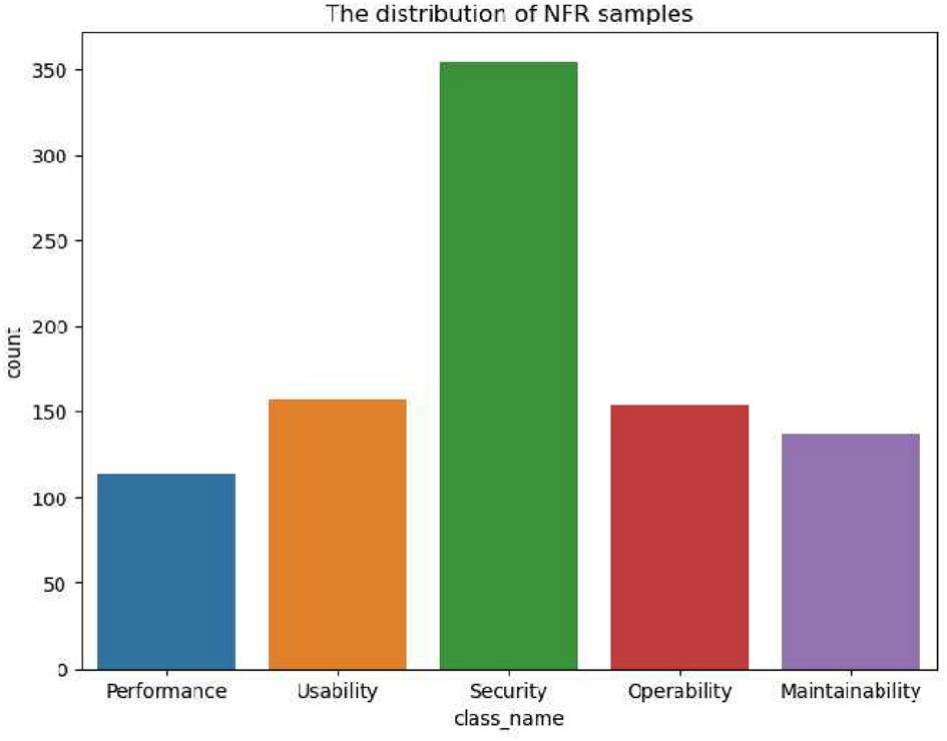
Categories-wise distribution of NFR in Data set.

Therefore, to reduce the negative impact of the imbalance dataset during training and learn the true underlying pattern, this study has used undersampling strategies and selected five categories of NFR. The composition of the resulting data set is depicted in Fig. 4. As a result, 914 NFRs consist of the following categories. Security (354), usability (157), operability (153), maintainability (137), and performance (113).

### Experimental setup

To experiment, first, mathematical and statistical tools are used to construct models. After creating the mathematical models, we put them into practice by implementing them using the Python programming language. This study used Python 3.11 and the Anaconda distribution as our primary coding environment for this study. Pandas library was used to handle and manipulate the corpus. Pandas have a wide range of features that can be used to enhance and analyze data as needed for this project.NLTK libraries were used to remove stop word lemmatization. To evaluate the accuracy and generalization of the model, the data was split into training testing data. To make the data compatible with algorithms, Sk-learn was used. Keras assisted in building up the DReqANN and DReqBiLSTM models. Before the training, the learning process was configured using compile() method. Where optimization, loss function, and matrices were specified. In this study, Adam optimizer and categorical_crossentropy were used as loss functions. Additionally, a list of matrices was also provided for the evaluation of the model. Training is an iterative process, so epochs, batch size, and call-back criteria were specified in the training of the models.

The proposed model DReqANN and DReqBiLSTM were trained with a limited dataset of 914 samples for 100 epochs. The training time for the DReqANN model was approximately 3.33 minutes on the configuration of a Google Collab NVIDIA V100 Graphics Processing Unit (GPU), and the training time of DReqBiLSTM was10.8 minutes under the exact hardware specifications. These training times provide computation efficiency and resource consumption of the proposed approach.

### Evaluation metrics

Various metrics are used in this study to evaluate the performance of the models in predicting the types of NFR. During the evaluation of the model, four different types of data are collected, including True Positive ($T_P$), True Negative ($T_N$), False Positive ($F_P$), and False Negative ($F_N$).

Accuracy 7 is dividing the number of accurate predictions by the total number of predictions, including both correct and incorrect predictions of the models.

7 $$\begin{aligned} {Accuracy} = \frac{TP + TN}{TP + FP + FN + TN } \end{aligned}$$

Precision (8) is the percentage of genuinely relevant instances (also known as true positives) out of all the examples predicted to fall into a particular class.

8 $$\begin{aligned} {Precision} = \frac{TP}{TP + FP} \end{aligned}$$

The recall (9) is the proportion of examples correctly predicted to be members of a class relative to all of the actual members.

9 $$\begin{aligned} {Recall} = \frac{TP}{TP + FN} \end{aligned}$$

The F1-score (10) is the mean average of precision and recall.

10 $$\begin{aligned} F1_{score} = 2\times \frac{Precision\times Recall}{Precision + Recall} \end{aligned}$$

To compute the micro average, let denote $y_l$ = set of predicted labels, $Y_l$ = Set of the actual label, $y = \cup y_l$, and $Y = \cup Y_l$. The Eq. (11) computes the micro average for precision

11 $$\begin{aligned} P(y,Y) = \frac{|y\cap Y|}{|y |} \end{aligned}$$

While Eq. (12) computes micro average for recall

12 $$\begin{aligned} R(y,Y) = \frac{|y\cap Y|}{|Y |} \end{aligned}$$

For macro averages, the Eqs. (13) and (14) are used for precision and recall respectively

13 $$\begin{aligned} P(y,Y)_{macro}= & {} \frac{1}{L}\sum _{l\in L} P(y_l, Y_l) \end{aligned}$$

14 $$\begin{aligned} R(y,Y)_{macro}= & {} \frac{1}{L}\sum _{l\in L} R(y_l, Y_l) \end{aligned}$$

For weighted averages, Eqs. (15) and (16) are used for precision and recall, respectively.

15 $$\begin{aligned}{} & {} P(y,Y)_{weighted} = \frac{1}{\sum _{l\in L}\mid Y_l \mid } \sum _{l\in L}{\mid Y_l \mid } P(y_l, Y_l) \end{aligned}$$

16 $$\begin{aligned}{} & {} R(y,Y)_{weighted} = \frac{1}{\sum _{l\in L}\mid Y_l \mid } \sum _{l\in L}{\mid Y_l \mid } R(y_l, Y_l)\end{aligned}$$

To measure sample averages for precision and recall Eqs. (17) and (18) are used.

17 $$\begin{aligned}{} & {} P(y,Y)_{samples} = \frac{1}{S}\sum _{s\in S} P(y_l, Y_l) \end{aligned}$$

18 $$\begin{aligned}{} & {} R(y,Y)_{samples} = \frac{1}{S}\sum _{s\in S} R(y_l, Y_l) \end{aligned}$$

### Results and discussions

This study aims to examine the impact of deep learning on the model’s performance while classifying multi-class non-functional software requirements. This study proposed two deep learning models, DReqANN and DReqBiLSTM, and compared the result with the shallower network. DReqANN performance was compared with a sample single-layer ANN model, and DReqBiLTM was compared with a single-layer BiLSTM model.

The result shows how better or poorer the introduced DReqANN and DReqBiLSTM models perform in processing requirements data to classify NFR. The result was analyzed to assess the impact of proposed deep learning models on the accuracy while performing multi-class classification of NFRs.The data set contained five classes of NFRs, and the models were trained on these five classes. Different metrics quantify the model quality, including precision, recall, F1-score, micro average, macro average, sample weighted average, confusion matrices, and accuracy. The measurement equations have been presented in the previous section of this study. Tables 3 and 4 compare the classification performance of our proposed models with baseline models. Thus, observing the individual class precision, recall, and F1- score provides a better understanding of the model’s performance. In Tables  3 and 4 bold value show better performance of a model on specific metrics to increase readability and interpretations of the results.

Table 3 compares the DReqANN model performance with the baseline ANN model. The result shows that precision for maintainability class is 100% in both models while recall of ANN is 67% and DReqANN Recall is 74%, F1-score of ANN is 80%, and DreqANN is 85%. Operability precision, recall, and F1-scores of ANN are 85%,74%, and 79%, while 81%,84%, and 83% for DReqANN. The performance result for ANN precision is 89%, recall 74%, and F1 score is 81%, while the precision of DReqANN is 90%, recall 83%, and F1-score is 86%.ANN precision for security class is 88%, recall is 90%, and F-1 score is 89%, while for DReqANN precision, recall and F-1 score is 89%. Usability precision for ANN is 89%, recall is 81%, and F1-score is 85%, while for DReqANN, precision is 90%, recall is 87%, and F-1 score is 89%.

**Table 3:** DReqANN VS ANN classifications report.

NFR Types	DReqANN classification report			ANN model classification report			Support
	Precision	Recall	F1-score	Precision	Recall	F1-score	
Maintainability	**1.00**	0.74	0.85	**1.00**	0.67	0.80	27
Operability	0.81	0.84	0.83	0.85	0.74	0.79	31
Performance	**0.90**	0.83	**0.86**	0.89	0.74	0.81	23
Security	0.89	**0.89**	0.89	0.88	**0.90**	**0.89**	71
Usability	**0.90**	0.87	**0.89**	0.89	0.81	0.85	31

The results show that an increase in precision decreases recall, while an increase in recall decreases precision. The optimal blend of both precision and recall is the F-1 score. A highly precise and recall model returns few results with high correct prediction. However, a model with low precision and high recall returns more results with highly incorrect predictions. Similarly, a high recall and low precision model returned all positive classes with highly inaccurate predictions. So, the ideal model has high precision and recall, returning more results with highly correct predictions. The result demonstrates that DReqANN performs better than ANN as they have high precision and recall and produce consistent results.

Table 4 compares the DReqBiLSTM model with BiLSTM models. The results demonstrate class-wise measurement of precision-recall and F-1 score of both models. The models were tested in each class for a better understanding of the performance of the models. Maintainability precision, recall, and F1-Score for DReqBiLSTM are 90%, 70%, and 79%, respectively. BiLSTM maintainability precision, recall, and F1-score are 71% for each matrix. The operability results for DReqBiLSTM precision, recall, and F-1 score are 83%, 77%, and 80%, while BiLSTM measurements are 74%, 67%, and 70%, respectively. Performance measurements for DReqBiLSTM are 91% precision, 87% recall, and 89% F-score, while for BiLSTM, the result is precision 88%, recall 91%, and F-1 score 89%. Security precision, recall, and F1-score for DReqBiLSTM are 88%, 85%, and 86%, and for BiLSTM 84%, 89%, and 86% respectively. Usability measurements for DReqBiLSTM are 73% precision,87% recall, and 79% F-1 score, while for BiLSTM, 83% precision, 65% recall, and 73% F-sore. By analyzing both models’ performance on individual NFR classes, the performance of DReqBiLSTM is slightly better than BiLSTM. The proposed deep learning model has achieved better results on the minimum data samples. The model has been trained on 713(80%) requirement documents while testing on 183(20%) requirement documents. It has been observed the models have promising results on such a small data set, so we can improve the results if we add more requirement documents for other classes.

**Table 4:** DReqBiLSTM versus BiLSTM classifications reports.

NFR types	DReqBiLSTM classification report			BiLSTM model classification report			Support
	Precision	Recall	F1-score	Precision	Recall	F1-score	
Maintainability	**0.90**	0.70	0.79	0.71	0.71	0.71	27
Operability	0.83	0.77	0.80	0.74	0.67	0.70	31
Performance	**0.91**	**0.87**	**0.89**	0.88	**0.91**	0.89	23
Security	0.88	0.85	**0.86**	0.84	0.89	0.86	71
Usability	0.73	**0.87**	0.79	0.83	0.65	0.73	31

The micro and macro average of the model has been presented in Table 5 and the best performing metric value is highlighted. Micro averages give importance to the number of samples in particular categories. If the support is high, those categories are more critical. The micro average is used in any situation and is the best suitable option when there are variations in class sizes. The micro average precision, recall, and F-1 values for DReqANN are 89%, 85%, and 87%, while for DReqBilLSTM, the values are 85%, 82%, and 83%, respectively. Micro average precision, recall, and F-1 score values for ANN are 89%, 80%, and 84%, while for BiLSTM, the values are 81%,80%, and 80%, respectively. In this study, we have considered the micro average F-1 score for model quality comparison. The results show that DRreqANN has the highest F-1 score as compared to the other three models, while DReqBiLSTM and ANN have equal scores with slight differences. BiLSTM micro average F-1 score is the lowest.

**Table 5:** Average micro and macro precision, recall and F-measure values.

Classifiers	Micro average			Macro average		
	Precision	Recall	F1-score	Precision	Recall	F1-score
DReqANN	**0.89**	**0.85**	**0.87**	**0.90**	**0.83**	**0.86**
DReqBiLSTM	0.85	0.82	0.83	0.85	0.81	0.83
ANN	0.89	0.80	0.84	0.90	0.77	0.83
BiLSTM	0.81	0.80	0.80	0.80	0.77	0.78

Macro average metric treats all classes equally and is a suitable choice in an imbalanced dataset. The macro average precision, recall, and F-1 values for DReqANN are 90%, 83%, and 86%, while for DReqBiLSTM, the values are 85%, 81%, and 83%, respectively. Macro average precision, recall, and F-1 values for ANN are 90%, 77%, and 83%, and for BiLSTM, 80%, 77%, and 78%. Marco’s average F-1 score values are selected for comparison of the models. According to the result, DReqANN performs better than other models, while DReqBiLSTM and ANN models have the same quality, while BiLSTM performs the worst.

Weighted averages consider the data split among various labels^18^. The weighted average represents the model performance; the high value shows the better performance of the model in the classification of NFR. The weighted average metric considers the relative importance of each class of NFR and then tacks the average of all values. Sample Average evaluates the model’s performance while considering the importance of all NFR classes equally. Weighted and sample averages for DReqANN, DReqBiLSTM, ANN, and BiLSTM are presented in Table 6 the bold value show the significant performance of specific model on respective matrices.The weighted F-1 score of DReqANN 87% is the highest, while the BiLSTM 80% score is the lowest. DReqBiLSTM and ANN have 83% and 84% weighted scores.

**Table 6:** Average weighted and sample precision, recall, and F-measure values.

Classifiers	Weighted average			Sample average		
	Precision	Recall	F1-score	Precision	Recall	F1-score
DReqANN	**0.90**	**0.85**	**0.87**	**0.90**	**0.85**	**0.87**
DReqBiLSTM	0.85	0.82	0.83	0.82	0.82	0.82
ANN	0.90	0.80	0.84	0.80	0.80	0.80
BiLSTM	0.81	0.80	0.80	0.80	0.80	0.80

**Figure 5:**
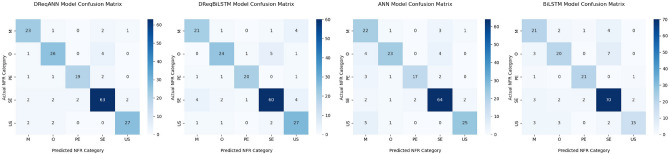
Confusion matrix of models.

The confusion matrix evaluates the proposed trained models, how successfully the models classify NFR, and where they make mistakes. The confusion matrix of all models is visualized in Fig. 5.

A confusion matrix provides more information than a simple accuracy metric. The diagonal line shows the true positive values of models. The X-axis shows the models’ predicted value, and the Y-axis represents the actual values. False negative values are the sum of corresponding rows except for true positive value, and false positive value for a class is the sum of the corresponding columns except True positives. A true negative value for classes is the sum of all rows and columns except the value of that class. The confusion matrix is easily understandable, and we can determine where the models confuse one class prediction with another. It also provided value for calculating the precision, recall, and accuracy of each model, either class-wise or of the whole models, and quantifying the quality of the models.

Accuracy is a straightforward metric that is easy to understand; however, the usefulness of this metric depends on the context to be considered while making decisions about the quality of the model. Table 7 describes training, validation, and overall model accuracy the bold value represents the better accuracy of the specific models. DReqANN has 84% accuracy, while DReqBiLSTM and ANN have the same 81% accuracy while the BiLSTM accuracy is 80%.

It has been observed that DReqANN with TFIDF has consistently performed better than DReqBiLSTM with sequential encoding. In the context of NFR classification, importance is given to the specific terms and their relevancy to particular classes. TFIDF effectively captures the significance of specific terms by assigning higher weights to frequent words in documents while relatively rare across the entire data set. The experimental results show that TFIDF effectively classifies NFR because the key terms play a crucial role in defining a particular class. On the other hand, sequential encoding effectively captures sequential dependencies and relationships between terms, leading to a semantic understanding of non-functional requirements.

**Table 7:** Classifier accuracy.

Classifiers	Training accuracy (%)	Validation accuracy (%)	Models accuracy (%)
DReqANN	97	**86**	**84**
DReqBiLSTM	**99**	83	81
ANN	98	83	81
BiLSTM	98	80	80

**Figure 6:**
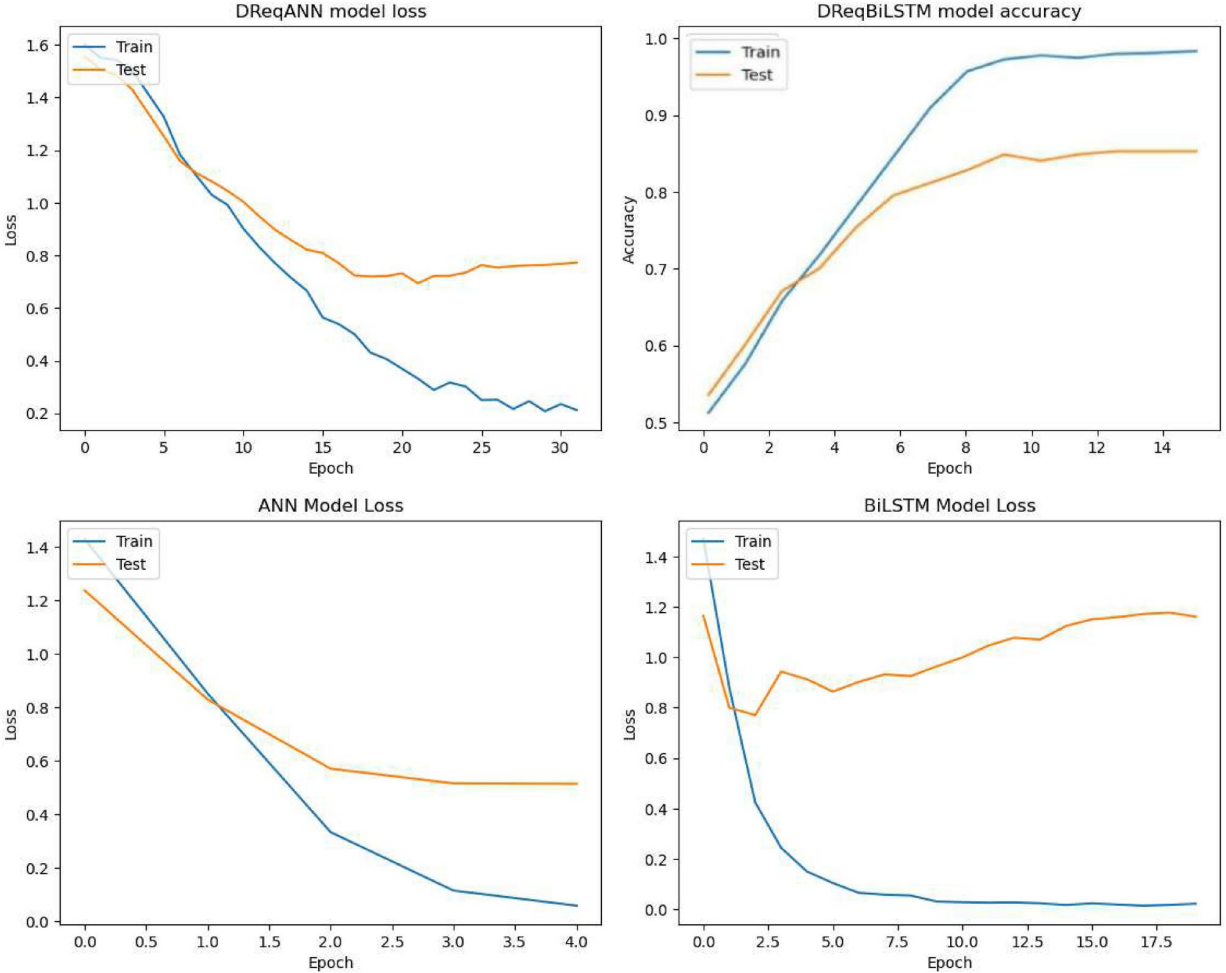
Models learning curve with accuracy-wise epochs.

The learning curve of all four models during the training, along with accuracy-wise epochs, has been shown in Fig. 6.

**Figure 7:**
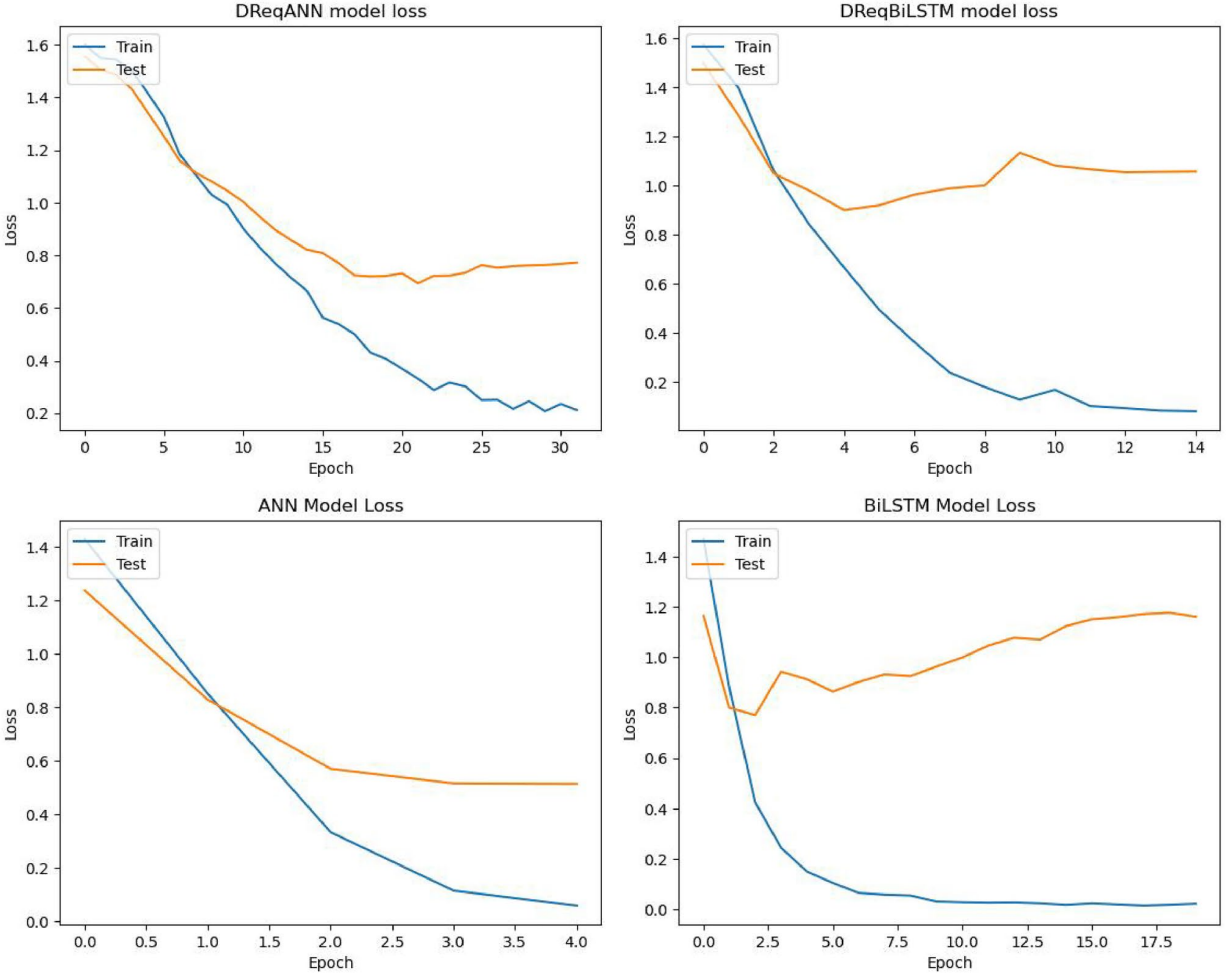
Models training and validation loss curve.

The training and validation loss of the model during training has been depicted in Fig. 7.

### Comparative analysis

A comparative analysis was conducted to highlight the advancement and improvement offered by our methodology compared to the state-of-the-art studies in the classification of NFR to assess the efficacy of the proposed approach.

Comparatively, an analysis of proposed approaches DReqANN and DReqBiLSTM with Hye et al. ^24^, Chatterjee et al. ^40^, Kumar et al. ^41^ and Baker et al. ^23^ has been presented in Table 8. The comparative analysis is based on precision, recall, and F1 score, which provide insight into the effectiveness of each approach. This comparison is based on the five most frequent classes of NFR and bold value show the better performance of proposed approach in compassion to the other approach to increase the readability of the results. The proposed approach DReqANN and DReqBiLSTM outperformed the other approaches even though they have used advanced language models like BERT. DReqANN and DReqBiLSTM provide a promising result in classifying NFR, while Heyet al  and Chatterjee et al.^40^ provide valuable insight into NFR predictions.

**Table 8:** Comparison with state of the Art approaches most frequent five NFRs.

Approaches	Maitainability			Operability			Performance			Security			Usability		
	Precision	Recall	F1-score	Precision	Recall	F1-score	Precision	Recall	F1-score	Precision	Recall	F1-score	Precision	Recall	F1-score
DReqANN(Proposed)	**1.00**	0.74	0.85	0.81	**0.84**	0.83	0.90	0.83	0.86	0.89	0.89	0.89	**0.90**	0.87	**0.89**
DReqBiLSTM(Proposed)	0.90	0.70	0.79	0.83	0.77	0.80	**0.91**	0.87	0.89	0.88	0.85	0.86	0.73	0.87	0.79
Hey et al.^24^	0.62	0.47	0.53	0.78	0.84	0.81	**0.92**	0.87	0.90	0.90	**0.92**	0.91	0.83	0.88	0.09
Chatterjee et al.^40^ (ISD1M4)	0.92	0.90	**0.91**	0.59	0.77	0.68	0.50	0.52	0.51	0.81	0.73	0.77	0.78	0.70	0.74
Indutrial Specific Dataset (ISD2 M4)	0.67	0.46	0.54	0.62	0.45	0.52	0.76	0.76	0.76	0.72	**0.94**	0.81	0.82	0.75	0.79
ISD3 M4	0.63	0.54	0.58	0.32	0.36	0.34	0.73	0.73	0.73	0.89	0.88	0.88	0.76	0.67	0.71
Kumar et al.^41^	**0.93**	0.82	0.87	0.75	**0.85**	0.80	0.91	0.91	0.91	**0.95**	0.90	0.92	0.66	0.85	0.75
Baker et al.^23^				0.85	0.81	0.82	0.86	0.79	0.81	0.82	0.83	0.85	0.90	0.75	0.83

Refer to Table 9 demonstrated the comparison of the proposed approach with fourteen (14) different state-of-the-art studies in the domain of NFR classifications. Each study has presented a different approach for the NFR classification using various ML algorithms. The evaluation is based on average precision, recall, and F1-score that shows the performance of the proposed approaches in the prediction and classification of NFR. The bold values show the better performance of the approaches for specific matrices. The proposed approach DReqANN and DReqBiLSTM exhibit higher average precision, recall, and f1 scores than most state-of-the-art approaches.

**Table 9:** Camprative analysis with an existing study on PROMISE NFR dataset.

Research work	Precision	Recall	F1-score
Cleland-Huang et al.^6^	0.24	0.76	0.36
Ormandjieva et al.^42^	0.72	0.72	0.70
Abad et al.^43^	0.90	0.91	0.90
Kurtanovic and Maalej^20^	0.78	0.81	0.77
Amasaki and Leelaprute^44^	0.77	0.67	0.68
Navarro-Almanza et al.^45^	0.81	0.78	0.77
Fong^46^	0.79	0.75	0.76
Baker et al.^23^	0.86	0.83	0.85
Gnanasekaran et al.^41^	0.83	0.85	0.83
Aldhafer et al.^47^	0.89	0.87	**0.87**
Li et al.^16^	0.87	0.79	0.83
Hey et al.^24^	0.86	0.88	**0.87**
Dekhtyar and Fong ^30^	**0.91**	0.91	0.91
Rahman et al.^31^	0.71	0.71	0.70
DReqANN (Proposed)	**0.90**	0.85	**0.87**
DReqBiLSTM (Proposed)	0.85	0.82	0.83

## Threats to validity

This section presents construct, internal, and external threats to the validity of the proposed approach.

### Construct validity

A standard experimental design and widely used metrics have been used to mitigate potential risks to construct validity. The models were trained and tested according to standard procedure. Precision, recall, and f-1 score metrics have been used to measure the quality of the model on the individual classes of NFR. Additionally, we have used macro average, micro-average, weighted average, and sample average for multiclass measurement. To mitigate the risk of learning rate selection that leads to instability or failure of the model during training, we have used techniques to reduce the learning rate when there is no change for a given number of training epochs. An excessive number of epochs in training leads to overfitting, while fewer epochs lead to model convergence problems. To mitigate this issue, we have used early stopping criteria that stop the training when the model starts overfitting without compromising the model’s accuracy. For replication of the study, we have used a fixed seed for random sample selection; the same 42 values used in this study as used by Dalpiaz et al.^48^.

### Internal validity

The ANN and BiLSTM-based models trained and tested in this study face threats to internal validity due to probabilistic mechanisms in dealing with multi-class classification problems. The neural network output is a real value that is converted through a transformation layer. The softmax function at the output layer converts them into values as probabilities between zero and one. The numpy argmax library picks the most significant probability as the observed class. This implies that the validity of classification based on the most significant probability without using weights or confidence intervals in such multi-category classification may threaten internal validity.

### External validity

The PROMISE_exp dataset that is used for experiments might be at risk of losing its external validity. The PROMISE_exp dataset is an expansion of the original PROMISE NFR dataset, and as a result, it shares the same quality problems with that dataset, as noted by Hey et al.^24^. Although the low-quality dataset may impact how well the different approaches performed in our experiments, this should not affect how generalizable these approaches are as we applied this dataset uniformly to all of them. Additionally, using the PROMISE NFR and PROMISE_exp datasets in research assessment enables us to directly compare outcomes to other methods because both datasets are well-known and acknowledged in the RE community. The results show that DReqANN produced promising results in classifying non-functional requirements. The dataset used in this study is relatively small and may only be representative of some fundamental requirements. Although it came from multiple projects, the properties may differ from actual projects. There must be more than one somewhat irregular data point to generalize the results^49^. Therefore, there is a need for more experimentation with a dataset having more requirements.

## Conclusion

The primary impediment encountered during the application of machine learning in the field of requirement engineering stems from the need for more high-quality data and the limitations imposed by the available supervised dataset. Traditional ML-based methods for NFR classification relying on handcrafted features show degraded performance when the feature space changes. However, deep neural networks select features automatically but require massive datasets. This study has proposed a comprehensive framework for classifying multiclass non-functional requirements, addressing the challenges analysts and developers face. The proposed framework eliminates the need for handcrafted features, providing a more automated and efficient approach to NFR classification. The main contribution of this study is the development of the DReqANN and DReqBiLSTM deep learning models tailored explicitly for NFR classification. These models incorporate advanced techniques such as word embedding, optimization and regularization techniques, and multi-layer architecture to classify NFR with limited datasets effectively. To validate the proposed framework, an experiment was conducted using publicly available data sets, ensuring the reliability and reproducibility of the results. The experimental findings demonstrate the effectiveness of the proposed framework in accurately classifying NFRs. The DReqANN model outperforms the other models in classifying NFR, achieving precision between 81% to 99.8%, recall between 74 and 89%, and F1-score between 83 and 89%.

Overall, this study significantly contributes to the field of NFR classification by offering a comprehensive framework, novel deep-learning models, improved accuracy, and empirical validation. However, it is vital to acknowledge the limitations of the proposed framework. The framework heavily relies on sequential preprocessing steps, and any inconsistencies in these steps can impact the accuracy of NFR classification. Additionally, the limited availability of training data in requirement engineering may lead to overfitting and poor generalization of unseen data. To overcome these challenges, the focus should be training transformer-based models on unsupervised software requirement corpus. The domain-specific pre-trained model will be used to predict and classify software requirements with the small available supervised dataset using transfer learning.

## Data Availability

The data is available at url: https://github.com/shanglapk/NFRDeepLearningFramework also cited the same in the reference list^32^.
